# Anesthesia

**Published:** 1915-07

**Authors:** Harold D. Henderson, Neil F. Jones


### ANESTHESIA.

READ BEFORE THE STUDENTS' DENTAL SOCIETY OF THE UNIVERSITY OF MICHIGAN, APRIL 20, 1915.

BY HAROLD D. HENDERSON AND NEIL F. JONES.

It is not definitely known at what period in the world's history anesthetics were first employed. However, it is recorded that the ancient Egyptians, Assyrians and Chinese were familiar with many vegetable substances capable of producing sedative and anodyne effects, and it is quite probable that these effects were taken advantage of in the practice of their crude surgery. The evolution of anesthetics has been very slow and it was not until the very close of the eighteenth century that our modern system of producing anesthesia began to make its appearance. The discovery of nitrogen, hydrogen, and oxygen, together with nitrous oxide, all within ten years, marked the commencement of a new era in chemistry and physics, and paved the way for the introduction into medical practice of a reliable system of inducing complete unconsciousness and of maintaining this state for a reasonable time without injurious consequences. The medical world gladly welcomed the idea of the therapeutic employment of vapors and gases. Thus we read that in 1795 a doctor employed ether as an inhalation for the relief of asthma. It is curious to note that there was a lapse of nearly half a century before these agents were turned to practical use in surgery. In 1842 Dr. Crawford W. Long, a country practitioner in the State of Georgia, administered ether with the object of producing insensibility to pain during a surgical operation which he performed. He appears, however, to have taken no steps to make his results known beyond the immediate circle in which he lived. It is not necessary to go into detail in this paper about the manner in which Horace Wells, an American dentist of his time, introduced nitrous oxide for producing anesthesia and which finally led up to the general use of anesthetics in the science of medicine and dentistry.

Anesthetics may be divided into two great classes, local and general, so classified because the first produces loss of sensation in a small portion of the body, or in the field of operation, whereas the second causes complete loss of sensation throughout the entire body. Local anesthetics are either injected, or applied on the surface, and affect the nerve endings with which they come in contact, while general anesthetics are inhaled and are carried by the circulation throughout the body. There are four methods employed to produce local anesthesia: namely, surface anesthesia, infiltration anesthesia, conductive anesthesia, and ganglionic anesthesia, respectively. Of these, the latter is not as a rule employed in dentistry.

There are four drugs which are employed to produce general anesthesia. They are chloroform, ether, nitrous oxide, and ethyl chloride. All of these produce anesthesia in about the same manner. The effect of the anesthetic is produced by the action of the drug upon the nerve cells of the body, thus bringing about a condition known as anesthesia. Function is temporarily depressed. It is not definitely known what process takes place in the loss of this function, or what combinations, if any, take place between the contents of the cell and the anesthetic. It is known, however, that the process of growth ceases, when tissues either in the vegetable or animal kingdom are anesthetized. The anesthetic reaches the cell through the circulation. When inhaled, it is taken into the lungs where it is taken up by the blood cells and carried to all parts of the nervous system. Anesthetics do not combine with the cell for a long period of time, but are quickly eliminated through the kidneys and the respiration, but fortunately the period of anesthesia can be controlled by the time and the amount of anesthetic administered. When an anesthetic is administered, the first effect is a slight stimulation of the nerve centers which produces an increased heart rate and causes the blood vessels to contract giving rise to an increased blood pressure. If too much anesthetic is administered at first, the mucous membrane may be irritated greatly, and spasms of the glottis be produced which may interfere with respiration. This tendency may be held in abeyance by the use of morphine and atropine. The period of stimulation causes an increase in all the bodily functions, especially the circulation, respiration, and sense of hearing. Next is noticed a slightly depressed condition and then the patient enters a period of excitement. This stage can be passed very quickly if enough of the anesthetic is administered and in many cases it is not noticed. It is characterized by great muscular activity on the part of the patient which may necessitate their being bound to the chair or operating table. This is the period of reflex activity. After the excitement stage, the patient enters into a sound sleep, or into the stage of anesthesia. It is in this stage that the operation is performed. When the reflex to the eye is lost, we know that the patient has entered this stage and it is by this symptom that we control the amount of anesthetic given. During this stage, great care must be taken to regulate the amount of anesthetic, and we should have on hand every thing needed to revive the patient in case of an accident. It is difficult to determine the relative safety of the anesthetics mentioned, but it is generally admitted that nitrous oxide with oxygen is the safest to use, because of fewer fatalities. Ether is fairly safe to use, only one death in about 15,000 administrations being recorded, while more deaths occur with chloroform than any of the others, one death in about 3,250 administrations. These figures are not altogether reliable, however, because accidents are due many times to carelessness and ignorance on the part of the anesthetist more than to the fault of the anesthetic. The elements of danger surrounding the administration of anesthetics may be classified as follows:

First. Ignorance, inexperience, and carelessness on the part of the operator.

Second. Length and duration of the anesthesia produced.

Third. Physical condition of the patient.

Fourth. Shock.

Of these, the first can be corrected if the anesthetist knows anesthetic symptoms and is a reliable person. The second is of small importance if the procedure is correct and the symptoms carefully observed. The third can be corrected before administration in most cases, and this leaves shock as the great factor in the successful administration of anesthetics. Shock is probably the factor that causes more accidents than anything else during anesthesia. Shock may be defined as a profound condition of depression. Many things may be done to reduce the number of deaths from shock, such as proper preparation of the patient, increasing their confidence, and care in administration. While accidents from shock may be reduced, they probably will never be eliminated. The general surgeon and dental surgeon administers an anesthetic primarily to prevent pain and avoid shock and secondarily to facilitate operating. Prior to the use of anesthetics, deaths were frequent in comparatively simple operations, due entirely to shock. For this reason it is that anesthetics are used. The dental surgeon also employs their use because shorter sittings are made possible which is beneficial to both himself and his patients. It also enables him to do a more thorough operation, and lastly, it should not be overlooked that it dignifies dentistry, elevating it to the plane of surgery, and increases the receipts of practice. If anesthetics are to be used in a practice, it becomes necessary for the dentist to carefully consider to whom it is safe to administer, and when they should be employed. Looking back over the last fifty years, at the progress that has been made in the line of anesthesia as compared to that which took place in all centuries previously, there is no doubt that a vast field is open to dentistry. The time will come when the infliction of pain in our profession will be an unknown factor.

